# Predicting price intervals under exogenously induced stress

**DOI:** 10.1371/journal.pone.0255038

**Published:** 2021-09-23

**Authors:** Steven Shead, Robert B. Durand, Stephanie Thomas

**Affiliations:** School of Accounting, Economics and Finance, Curtin University, Bentley, WA, Australia; Centre for Economic and Regional Studies of the Hungarian Academy of Sciences, HUNGARY

## Abstract

We present an experimental protocol to examine the relationship between exogenously induced stress and confidence in a setting applicable to financial markets. Confidence will be measured by a prediction interval for a one period ahead price forecast, based on a series of 100 previous prices; narrower (wider) prediction intervals will be indicative of greater (lower) confidence. Stress will be induced using the Cold Pressor Arm Wrap, a variation of the Cold Pressor Test. Risk attitudes, and personality traits are also considered as mediating factors.

## Introduction

We present an experimental protocol to test if exogenously induced stress is associated with confidence. If there is an association, we will consider whether stress has a negative or positive relationship with confidence. Our protocol also proposes that we examine the mediating roles of participants’ risk aversion (measured using the Holt and Laury [1] risk assessment task) and personality traits (measured with the ‘Big 5’ personality inventory [2]. Furthermore, as our experimental design has participants responding to patterns (in our case, prices), we present hypotheses about how uncertainty (volatility) and perceived direction of trends may affect any relationship between stress and confidence.

The protocol proposes that stress is induced exogenously (discussed in the stress section of this protocol). The physiological changes that the treatment induces have been shown to cause cognitive adjustments associated with stress. Stress can be both exogenous and endogenous. In financial markets, the setting which motivates our experiment, investors respond efficiently (that is, quickly) to exogenous stimuli (information). Investors responses to such exogenous events will, in turn, affect their stress [3]. These responses bring about revisions of expectations and, consequentially, can generate trading and price changes [4, 5]. Such exogenous stimuli can include unexpected events such as take-over announcements, surprise earnings (either better or less than the market expects) or new products. While unexpected, such events are familiar to the market and the broad responses are well understood by market participants and academics; the study of the effects of these events on investors’ wealth has a specific methodology (see [6] for a thoroughgoing introduction to the event-study methodology). Occasionally, the market has to deal with less familiar events. [7] document EntreMed’s dramatic price rise (and price rises in other biotechnology firms) following a news report of it having a cancer curing product; and the subsequent fall in its price when this was found not to be so. These shocks are exogenous as they are independent of the market and could occur regardless of firms being listed. A portion of the response to such shocks may potentially be attributable to individuals’ reaction to stress, while other portions may be attributable to behavioural phenomenon such as herding or fear or missing out. These exogenous events create abnormal returns but are independent and, typically after a relatively short period during which the information is discounted, returns resume their random walk. As we discuss below, we use an exogenous stressor to generate physiological changes that can induce cognitive adjustments, thereby isolating the impacts of such adjustments at the individual level. This allows for the identification of the impact of the physiological response to *stress itself* upon individuals’ forward predictions *independent* of the effects of market feedback and other behavioural phenomenon that typically accompany market events.

While our analysis may be generalisable to exogenous stress and confidence in a range of domains, our study is motivated by the stress experienced by participants in financial markets and the role of confidence. In particular, miscalibrated confidence—overconfidence—has been the focus of considerable attention in the behavioural finance literature. To our knowledge stress per se, and the interaction of stress and confidence, has not been considered. Our experimental design, discussed below, has participants forecasting the upper and lower bounds of the next price on a screen not unlike those available to institutional and individual investors. When we discuss the experimental design, we operationalise the concept of confidence by analysing the difference between the upper and lower bounds of participants’ forecasts. A higher (lower) bound range indicates lower (higher) confidence. Participants are incentivised to be truthful (similar to [8]) and there is a unique optimal choice which maximizes returns.

The structure of this paper is as follows. We will briefly review the literature on the role of confidence in financial decision making, as well as the role of stress. Next, we introduce the experimental design, and parameterisation. Throughout, we present sixteen hypotheses which we aim to test. The section “Assessment of hypotheses” describes the approach to the analysis. Finally, we conclude, reiterating the potential contribution of this work to the literature.

## Confidence

Confidence plays an important role in financial decision making. [9] argue that short-term confidence is an important determinant of fund manager survival in financial markets, postulating that portfolio managers retain more clients when they are perceived to be more confident. [10] offer an evolutionary perspective, in a model of competition in which more confident predatory species take more risks and, all things being equal, benefit through having a higher chance of survival. Confidence is not universally advantageous, however; the wrong level of confidence can have detrimental consequences. [11] suggest that over-confidence, for example, is associated with excessive trading which damages investors’ wealth, while [12] find that under-confidence is associated with less frequent trading.

Investing involves committing funds, and deferring consumption, in anticipation of positive returns, and increased consumption, in the future. Investors must have some belief about the future and forecasts can be critical in forming these beliefs. Sell-side analysts provide earnings and price forecasts to investors, the “buy side”. Studies of these forecasts have found evidence of the importance of both confidence and stress in analysts’ behaviors yet, to our knowledge, the experimental design presented in this protocol is the first to consider any relationship between confidence and stress. [13] find that confidence has a positive association with forecasts that are different from the prevailing consensus (“anti-herding”); despite the potential rewards for standing out [14] estimate rewards of between $9 and $168 million for analysts in the “bulge bracket“), analysts tend to herd when forecasting [15]. Such herding is not consistent with providing value to clients, but it is consistent with analysts experiencing stress [16, 17].

Confidence can be influenced by factors such as information about a task, familiarity with a task and feedback which confirms one’s own beliefs. [18] suggest that increased information will increase confidence: individuals become more confident in their answers and less willing to admit their mistakes. These authors also observed that having more practice with a task increased confidence. However, these factors can also lead to excessive confidence. [19] for example, discusses the way in which confirmation bias can contribute to over-confidence. Another source of over-confidence that [19] identifies is the misinterpretation of information related to an investment, leading to poor trading decisions.

It is important that investors have the right level of confidence when considering where to allocate their funds. The wrong level of confidence can result when individuals misperceive the world around them. The difference between an individual’s perception of a probability and their belief in the correctness of that perception is commonly referred to as *miscalibration* [20]. Individuals who are *over-confident* have stronger beliefs about the correctness of their perception, constructing prediction intervals that are too narrow, while individuals that are *under-confident* have weaker beliefs (or have more vague perceptions about a probability), and will tend to construct prediction intervals that are wider than necessary. [21] found that investors with stronger beliefs about the precision of their forecasts tended to optimistically predict greater levels of future stock performance which resulted in income losses when true returns were realised.

There is some evidence to suggest that over-confidence is a consistent individual characteristic. [22] found significant correlation of within-participant over-confidence across domains, comparing reported confidence and performance on questions with existing correct answers against asset forecasting tasks which lack correct answers *a priori* (and therefore have a less clear relation to under- or over- confidence). Under-confidence may be less clear, however. [23] found that some investors rely more heavily upon intuition when predicting future asset movements in the stock market. A participant who relies on intuition to produce a prediction interval in our experiment might select a wider interval. [24] found that participants had more confidence in answers to knowledge-based Math and English questions and less confidence in answering an intuition-based question, despite being more accurate in answering the intuition based question.

In this study participants will submit prediction intervals which define the size of the payoff that is received if a randomly generated price falls within the submitted interval. As will be shown, a payoff maximising interval exists for any chosen lower bound of the prediction interval if participants accurately interpret stochastic movements in a series of prices. Participants who submit wider intervals than the payoff maximising level forgo earnings in order to increase the probability of being correct and are classed as under-confident. Participants who submit narrower intervals than the payoff maximising level reduce the probability of being correct while increasing their payoff and are classed as over-confident. We turn now to the influence of stress on decision making.

## Stress

[25] define stress as “an adaptive reaction to an adverse stimulus or a situation” and outline three characteristics that are associated with acute stress: sympathetic nervous system activation, endocrine response (an increase in cortisol), and cognitive adjustment, a process consistent with [26]’s description of sympathetic nervous system activation. S1 Fig outlines this path.

The Cold Pressor Test (CPT) is frequently used to trigger a physiological stress response and, in our proposed experiment, the CPT will be used to induce exogenous stress. Participants exposed to the CPT submerge a hand in a cold water bath, which causes reversible pain and activates the sympathetic nervous system [27–29]. The Cold Pressor Arm Wrap (CPAW), which we will use, achieves the same effect [30], but is more suited to be implemented in a computer lab setting as a cold ice pack is used, instead of a cold water bath. Evidence that the CPT has the ability to induce a physiological state of stress can be found in [28, 31, 32]. [33] also show the clear impact of the CPT on cortisol levels.

The impact of heightened cortisol on cognitive processing can be observed in the decisions made by participants. [34] use the CPT to investigate the impact of stress on financial decisions. They do not, however make the distinction between exogenous and endogenous stress, as we make in this protocol. Participants were required to make a series of binary lottery-like decisions which were framed as either losses or gains but which were equivalent in expected pay-offs. On average, preferences to avoid risks when losses were involved and to prefer risks when gains were involved were accentuated under stress. [33] also used the CPT and found that participants’ decisions remained rational under stress in a task that examined the Generalized Axiom of Revealed Preference. This contrasting result could suggest that the domain of the task is relevant, and that some tasks are less sensitive to stress. Our first hypothesis is thus:

**Hypothesis 1 (Prediction intervals are not influenced by exogenous stress)**
*Rejection of Hypothesis 1 will be taken as evidence that exogenous stress in fact does influence the construction of prediction intervals*.

## Risk attitudes and personality traits

In a study that examined the interaction of over-confidence and investor risk attitudes, [35] found over-confidence to be decreasing with respect to levels of risk aversion. [20] suggest that more (less) risk averse participants may construct wider (smaller) prediction intervals. In our study, participants will complete the Holt and Laury risk assessment task [1] to enable a test for the role of risk attitude in the formation of prediction intervals. This leads to our next general (null) hypothesis:

**Hypothesis 2 (Prediction intervals are not sensitive to individual level of risk aversion.)**
*Rejection of Hypothesis 2 would suggest a link between risk aversion and prediction interval size*.

It could be the case that more risk averse individuals respond to stress differently than those who are less risk averse. Thus we hypothesise

**Hypothesis 3 (Responses to stress are not affected by individual levels of risk aversion)**
*Rejection of this hypothesis would suggest that risk attitudes interact with exogenous stress to impact upon prediction interval formation*.

[36] found that three of the Big Five personality traits (Negative Emotion, Extraversion, and Agreeableness) were correlated with overconfidence in financial decisions. [37] found ‘Extraversion’ to be associated with *over-confidence* but not with confidence, and ‘Openness’ to be associated with confidence but not over-confidence. We hypothesise that

**Hypothesis 4 (Prediction intervals are not sensitive to personality traits)**
*Rejection of this hypothesis would suggest that personality plays a role in the formation of prediction intervals*.

Similar to risk aversion, it may be the case that personality interacts with exogenous stress leading to different responses. Thus, we also hypothesise:

**Hypothesis 5 (Responses to exgenous stress are not affected by personailty traits)**
*Rejection of this hypothesis would suggest that personality plays a role in the response to exogenous stress and affects prediction intervals*.

## Experimental design

The main outcome of interest in this study is the size of the interval within which participants predict an unknown one-period-ahead price. Participants will be shown a total of 30 pre-generated price series in a pre-determined sequence which is repeated once for a total of 60 prediction interval choices. The CPAW will be used to induce a physiological stress reaction in one of the sets of 30 choices. Each participant will experience 30 consecutive decisions with stress and 30 consecutive decisions without stress. In order to maintain comparability in the timing, flow of the experiment, and the level of stress response, participants will be asked to apply an arm wrap for 2 minutes at the start of each set of 15 decisions. Under the unstressed condition the arm wrap will be at room temperature, while under stress the arm wrap will be between 0 to 5°C upon application. [28] suggest that the cortisol level response to the CPT lasts for 15 minutes following immersion of one hand in cold water for 2 minutes. We estimate that participants will take roughly one minute to evaluate and submit each price prediction interval, and thus to maintain a relatively constant level of cortisol we will administer the CPAW after each set of 15 decisions. At the same frequency the room temperature CPAW will be applied to control for any effects of distraction or other effects attributable to the procedure itself.

This design was chosen to enable within-subject comparisons of the response to exogenously induced stress with otherwise identical decisions. It would be unusual for a participant to recall a sequence of 30 elements, so from the participant’s perspective, the repetition should not be noticeable. Half of our participants will experience the stress condition for the first 30 decisions followed by 30 decisions without stress, with the order reversed for the other half. This is to provide a control for learning effects, decision fatigue, or persistence of the stress response.

To control for the possibility that prediction intervals are influenced by the order of presentation of the price series, we also present two different sequences of 30 decisions, presented in Table 1. These sequences were developed by rearranging ten unique pre-determined price series, which is presented in S2 Fig. This arrangement provides several direct comparisons of prediction intervals which occur at the same point in the sequence, but have followed a different history. For example, at periods 7, 14, 22, and 29, all participants submit decisions on the same price series. The details of the ten price series are discussed in the section “Price series parameterisation”.

**Table 1:** Sequence of presentation of price series.

Period	Sequence A	Sequence B
1	I	I
2	J	J
3	E	G
4	F	H
5	B	D
6	A	C
7	I	I
8	J	J
9	B	A
10	F	E
11	J	I
12	H	G
13	D	C
14	A	A
15	B	B
16	I	I
17	J	J
18	G	E
19	H	F
20	D	B
21	C	A
22	I	I
23	J	J
24	A	B
25	E	F
26	I	J
27	G	H
28	C	D
29	A	A
30	B	B

Feedback can confound the main variable under investigation, in this case, confidence. [38] found that feedback that contradicted participants’ estimates resulted in lower levels of confidence but feedback that confirmed participants’ estimates lead to overconfidence. In order to isolate the effect of exogenous stress from feedback a possible source of (exogenous stress) on prediction intervals (confidence), feedback on the success or failure of the submitted intervals will be withheld until the end of the experiment.

Table 2 summarizes the design. [39] suggest that 30 participants in each cell will be sufficient when the distribution of outcomes are unknown prior to the experiment. A power analysis for paired t-tests (within subject tests) supports a similar number: an effect size of 0.5 (Cohen’s-d) with 5% significance and 80% power results in 33.37 participants in each cell. However, this analysis is limited only to the case in which a simple (single) hypothesis, such as Hypothesis 1, is tested. In order to investigate all 16 hypotheses proposed in this study a multiple regression framework approach will be taken and is discussed in greater detail in the hypothesis testing section below. From this approach, Cohen’s F^2^ effect size is used in the assessment of statistical power [40, 41]. Each participant represents an independent experimental unit, and pooling the four experimental cells of 30 participants results in a complete sample size of 120. With this sample size, and setting the power to 80%, the significance level to 5%, and the number of individual variables in the model to 18, the minimum effect size that can be detected is 0.067; which is considered to be a small effect size. Thus, our selected design will have power of at least 80% when jointly testing for the relevance of the factors that may influence prediction interval ranges with controls for risk attitudes and personality traits. We will thus recruit 30 participants per cell in Table 2. In total, 120 participants will be recruited using an existing participant pool at the University Experimental Economics Lab. The pool includes students and the wider community. Participants with contraindicated health conditions will be excluded from participation. The experiment has been approved by the Curtin University Human Research Ethics Committee (HRE 2020–0415). Participants will be paid in accordance with standard experimental lab procedures (in cash privately at the end of the experiment). Participants are randomly allocated to computer workstations upon arrival at the lab (after completing an information and consent forms) and responses are identified only with an anonymous code.

**Table 2:** Experimental design and number of participants.

	Stress in first 30 periods	Stress in second 30 periods
Sequence A	30	30
Sequence B	30	30

## Payoff structure and model of prediction interval choice

A potential concern is that participants might select wider bands than they would truthfully have chosen to ensure the likelihood of a payout. For example, participant X might make a prediction interval wider than she truthfully believes if a payment is contingent on simply being within a certain band. In this study, we use a payoff structure that incentivises participants to tell the truth about their beliefs. In doing so, we closely follow [8]. To ensure that participants understand this mechanism we have included a set of comprehension questions following the instructions (included in the S1 Appendix). We also examine participants’ risk preferences in making these decisions by considering whether their decisions are related to their Holt and Laury [1] risk attitude score. As well, we consider the role of personality using the ‘Big 5’ Personality traits.

Eq (2) outlines the payoff structure which is dependent upon experiment parameters *M* (the maximum allowable range for a prediction interval) and *B* (the payment earned if the prediction interval contains the unknown next period price). Payoffs are defined as a proportion of the total allowable range. Participants thus trade-off the *probability* that the next price will be contained within the submitted interval against a *payoff* which is decreasing in the size of the submitted interval. Each price series will be presented to participants within an identical allowable range between 0 and 500, thus *M* = 500. *B* is set to be 100 ‘lab points’ with 100 points being converted to $0.70 in local currency at the end of the experiment and paid to each participant in cash.

The choice of a prediction interval for the one period ahead price is a trade-off between the probability that the next price falls within the interval and the return to the decision maker for being correct. The price in period *t*, *P*_*t*_, is the result of a pre-determined process which we define later in Eq (5).

Let the prediction interval be defined by the lower and upper bound, *P*_*l*_ and *P*_*u*_, respectively. The probability that *P*_*t*_ is within a prediction interval is thus:

$$\begin{array}{c} Pr\left(P_{t}\in \left[P_{l},P_{u}\right]\right)=\int_{P_{u}}^{P_{l}}f\left(x\right)dx\equiv \Phi \left(P_{u}\right)-\Phi \left(P_{l}\right). \end{array}$$ (1)

Payoff *B* is earned if *P*_*t*_ ∈ [*P*_*l*_, *P*_*u*_], and is inversely proportional to the size of the prediction interval:

$$\begin{array}{c} \begin{array}{c} \text{payoff}=\{\begin{array}{cc} (1-\frac{P_{u}-P_{l}}{M})B\; & \text{if}\;P_{t}\in \left[P_{l},P_{u}\right]\\ 0\; & \text{otherwise} \end{array} \end{array} \end{array}$$ (2)

with parameter *M* being the maximum possible range the interval can take on. The decision maker’s task is thus to choose the prediction interval in order to maximise the *expected payoff*:

$$\begin{array}{c} E\left(\text{payoff}\right)=(\Phi \left(P_{u}\right)-\Phi \left(P_{l}\right))(1-\frac{P_{u}-P_{l}}{M})B \end{array}$$ (3)

Letting *P*_*u*_ − *P*_*l*_ = *δ* and taking the derivative of E(profit) there is a unique optimal prediction interval, δ*, defined for any choice of a lower bound, *P*_*l*_:

$$\begin{array}{c} \delta^{*}=M-\frac{\left(\Phi \left(P_{l}+\delta \right)-\Phi \left(P_{l}\right)\right)}{\Phi^{\prime }\left(P_{l}+\delta \right)} \end{array}$$ (4)

Further details can be found in the S1 Appendix. This result leads to our next general hypothesis:

**Hypothesis 6 (Prediction intervals maximise expected returns ($\delta_{i,t}=\delta_{i,t}^{*}$) for each participant, *i*, in each period *t*.)**
*Rejection of this hypothesis would imply that the formation of confidence intervals is not consistent with payoff maximisation*.

We extend this hypothesis to incorporate of the potential effect of exogenous stress on payoff maximisation:

**Hypothesis 7 (Payoff maximisation is not affected by stress $\left(\delta_{i,S}-\delta_{i,S}^{*})=(\delta_{i,N}-\delta_{i,N}^{*}\right)$ for each participant *i* under conditions with stress, *S*, and no stress, *N*.)**
*Rejection of this hypothesis would suggest that exognousstress impacts upon the process of optimisation*.

The experimental interface is implemented using oTree [42]. S3 Fig illustrates the layout of the main decision screen. Participants are presented with a series of 100 prices and asked to place their lowest prediction value in the box under the sentence “what will be the lowest next price?” and their highest prediction value in the box under the sentence “what will be the highest next price?”. A button is provided to assist with calculation of the payoff that will occur if the next price falls within the submitted interval. The calculator feature is provided in order to ensure that the payoff mechanism is clear and thus decisions are reflective of individual participants’ preferences and not misunderstanding of the payoffs. The calculator can be used repeatedly and is provided on each decision screen. When the participant is satisfied with the interval, the red button is clicked to submit and the next price series is shown.

The Holt and Laury [1] risk assessment task will be completed before any prediction interval decisions. Any potential priming effects that might occur as a result of asking participants to choose among gambles before submitting price interval predictions would not be inconsistent with our research objectives. The Big Five personality trait questionnaire [43–45] will be administered after all prediction interval decisions have been submitted. The placement of this questionnaire at the end of the experiment is to avoid potentially priming participants into thinking about their personality when making prediction interval choices, and because this is a longer questionnaire which might lead to early decision fatigue.

If exogenous stress or individual characteristics influence the payoff maximisation process, difference *δ* − δ* will be affected. Thus we also hypothesise, similar to Hypotheses 2–5, that the optimisation of prediction intervals is unaffected by individual levels of risk aversion, personality traits or the combination stress and individual characteristics.

**Hypothesis 8 (Payoff maximisation is not sensitive to risk attitude $(\delta_{RA}-\delta_{RA}^{*})=0\forall RA$, such that *RA* is the level of risk aversion.)**
*Rejection of this hypothesis would suggest that risk attitude affects the payoff maximisation process*.

Combining this with stress,

**Hypothesis 9 (Payoff maximisation is unaffected by the interaction of exogenously induced stress and level of risk aversion $(\delta_{RA,S}-\delta_{RA,S}^{*})=(\delta_{RA,N}-\delta_{RA,N}^{*})\;\forall RA$ under conditions with stress, *S*, and no stress, *N*.))**
*Rejection of this hypothesis would suggest an interaction between exogenously induced stress and risk attitude which impacts upon the payoff maximisation process*.

With respect to personality traits we hypothesise:

**Hypothesis 10 (Payoff maximisation is not sensitive to personality traits. $(\delta_{\text{Big5}}-\delta_{\text{Big5}}^{*})=0\;\forall \;\text{Big5}$, where Big5 refers to the Big 5 personality traits.)**
*Rejection of this hypothesis would suggest that personality traits affect the payoff maximisation process*.

Finally, we combine to investigate the potential interaction with stress:

**Hypothesis 11 (Payoff maximisation is unaffected by the interaction of exogenously induced stress and personality traits $(\delta_{\text{Big5},S}-\delta_{\text{Big5},S}^{*})=(\delta_{\text{Big5},N}-\delta_{\text{Big5},N}^{*})\;\forall \;\text{Big5}$ under conditions with stress, *S*, and no stress, *N*.)**
*Rejection of this hypothesis would suggest an interaction between exogenously induced stress and personality traits which impacts upon the payoff maximisation process*.

## Price series parameterisation

For this experiment we have designed 10 unique price series that contain 100 prices using the pre-specified structure of Eq (5). This structure allows us to specify the direction and intensity of trends, as well as the variance of the prices in a controlled manner.

$$\begin{array}{c} P_{t}=P_{0}+\beta_{1}t+\beta_{2}P_{t-1}+\epsilon_{t},\;\text{s.t}\;\epsilon ∼N\left(0,\sigma^{2}\right) \end{array}$$ (5)

*P*_0_ is the initial price of the time series, *β*_1_ a parameter defining the general slope of the series, and *P*_*t*−1_ the price in the previous time period. *β*_2_ is a fixed parameter defining the persistence of previous prices. *P*_*t*_ is defined by a random error term, *ϵ*_*t*_, which is normally distributed with mean 0 and variance *σ*^2^.

The price series presented to participants are derived from Eq (5). *ϵ*_*t*_ is drawn from one of two distributions with differing variances. Two sets of *ϵ*_*t*_ were drawn, one from each distribution respectively and used to create the prices series shown to participants. The price series presented are therefore identical and for any two prices series sharing the same standard deviation parameter, the optimal intervals are equivalent. Participants will have to respond to the upward and downward slopes in a statistically distinctive manner in order for our analysis to find differences between the slope scenarios. This approach to constructing price series has not commonly been done in research. [21, 46–48] for example, use time series collected from securities in financial markets.

In constructing the price series presented to participants, we have been mindful to produce price series which realistically appear like those seen in markets. However, we have intentionally designed the price series imposing a strict parameterisation for each element in Eq (5). *β*_1_, which defined trends, takes on two values (for strong or weak trends) and we use the positive and negative of these value to consider upward and downward trends of the same intensity. We also include series without any trends by setting *β*_1_ equal to 0. *P*_0_ is one of three values, selected to ensure that each series ends at approximately the same point in the middle of the allowable range. The *ϵ*_*t*_ term is normally distributed with a mean of 0 and two different standard deviations. Two pre-generated random draws were applied to construct the price series. Table 3 summarises the parameterisation. This structured approach allows for a parallel investigation of the sensitivity of prediction intervals to volatility and trends, and strengthens the ability to test hypotheses surrounding these variables.

**Table 3:** Properties of the generated price series.

Price Series	Trend Direction (*β*_1_ > 0, *β*_1_ < 0 or *β*_1_ = 0)	Trend Intensity (*β*_1_)	*σ*
A	Upward	Weak	Low
B	Upward	Weak	High
C	Downward	Weak	Low
D	Downward	Weak	High
E	Upward	Strong	Low
F	Upward	Strong	High
G	Downward	Strong	Low
H	Downward	Strong	High
I	Flat	Flat	Low
J	Flat	Flat	High

Note:

Exact parameter values are available from the corresponding author upon request.

Characteristics of the price series *P*_*t*_ may influence the size of prediction intervals, *δ*. [48] observed a tendency for participants to follow trends when presented with downward or upward sloping time series. However, [21] observed both trend-following and trend-bucking choices among participants exposed to a downward sloping trend. Thus, we also hypothesis:

**Hypothesis 12 (Prediction intervals are not responsive to volatility, ($\delta_{\sigma_{j}^{2}}=\delta_{\sigma_{k}^{2}}\;\forall \sigma_{j}^{2}\neq \sigma_{k}^{2}$))**
*Rejection of this hypothesis suggests that prediction intervals are responsive to the degree of variability in the price series*.
**Hypothesis 13 (Prediction intervals are not responsive to the direction of the trend, $\delta_{\left(\beta_{1}<0\right)}=\delta_{\left(\beta_{1}=0\right)}=\delta_{\left(\beta_{1}>0\right)}$)**
*Rejection of this hypothesis suggests that prediction intervals are responsive to the direction of the trend of the price series*.
**Hypothesis 14 (Prediction intervals are not responsive to the intensity of the trend, $\delta_{\left(\beta_{n}\right)}=\delta_{\left(\beta_{m}\right)}\;\forall \beta_{n}\neq \beta_{n}$)**
*Rejection of this hypothesis suggests that prediction intervals are responsive to the intensity of the trend of the price series*.

Exogenous stress may also influence the way in which characteristics of the price series are interpreted, thus:

**Hypothesis 15 (Prediction intervals are not responsive to the interaction of exogenous stress and volatility, ($\delta_{\sigma_{S}^{2}}=\delta_{\sigma_{N}^{2}}$ under conditions of exogenous stress *S* and no stress *N*.)**
*Rejection of this hypothesis would suggest an interaction between exogenous stress and the variability of the price series*.
**Hypothesis 16 (Prediction intervals are not responsive to the interaction of stress and direction or intensity of the trend of the price series, ($\delta_{\beta_{S}}=\delta_{\beta_{N}}$ under conditions of exogenously induced stress *S* and no stress *N*.)**
*Rejection of this hypothesis would suggest an interaction between exogenously induced stress stress and the direction or intensity of the trend of the price series*.

Rejection of any of Hypotheses 12–16 accompanied by a sufficient standardised effect size would imply that the structure of the price series influences prediction interval choices, and/or a potential role for stress to influence the interpretation of volatility, trend direction, or intensity of trends in the price series.

## Assessment of hypotheses

The core research question, the relationship between exogenously induced stress and confidence is addressed in Hypothesis 1. We will test the key hypothesis using a within subject comparison, examining the same participant’s prediction intervals with and without stress using standard paired Hypothesis test procedures (as appropriate to the data). Rejection of the null Hypothesis will provide evidence that levels of confidence are affected by exogenous stress.

Hypotheses 2–16 (in addition to confirming Hypothesis 1) will be tested under a multiple regression framework, with explanatory variables including the order of the stress treatment (first or second), sequence of price series (A or B), slope (weak, strong or flat), direction (positive, negative or flat), volatility (low or high), the Holt and Laury risk assessment (the level of risk aversion determined by the switch point in a multiple price list), and each of the Big Five personality traits (Extraversion, Openness, Agreeableness, Conscientious and Negative Emotion). Additionally, an examination of the interaction effects of stress on certain variables will be conducted as outlined in Hypotheses 9, 11, 15 and 16. Period number and participant random effects will be included to control for the potential impact of time (due to for example fatigue or desensitisation) and individual differences. Graphical representations of the data will be used to support the analysis.

Hypotheses 2–5 will examine the role of risk attitude and Big 5 personality traits for potential mediating effects on prediction intervals. Hypotheses 6–11 test for impacts on profit maximisation (i.e. an alternative outcome measure). Hypotheses 12–16 examine the impact of characteristics of price series on prediction intervals. To quantify substantiveness results will be accompanied by standardized effect sizes.

## Conclusion

To the best of our knowledge this is the first experimental study of the role of exogenously induced stress in predicting future stock price movements. In particular, we seek to understand how exogenously induced stress and confidence may interact in financial decisions which involve forecasting future stock prices. Our work is motivated by the importance of confidence for participants in financial markets and that activity in these markets may be influenced by exogenous stress.

In the proposed experiment, confidence will be proxied though prediction intervals obtained from participants’ forecasting the range of a series of prices. A smaller (wider) prediction interval will be taken as a sign of greater (lower) confidence. A total of 120 participants will each produce 60 prediction intervals: 30 without stress and 30 with stress, with stress induced using Cold Pressor Arm Wrap (CPAW), a tractable variation of the Cold Pressor Test (CPT).

The price series viewed by our participants are constructed to allow us to test whether there is an association between exogenously induced stress and confidence under price series with varying trend directions and intensities, as well as volatilities. This study will also examine the relationship of risk attitudes and personality traits for their potential mediating role in the impact of stress on confidence.

While confidence has been studied using market proxies, it is difficult to conceive how the interaction of confidence and exogenous stress could be considered outside the laboratory. Examining these two variables in a laboratory setting may provide instructive lessons in how financial markets behave. It has been established that excessive levels of confidence are detrimental to investors. Evidence of the effects of stress *per se* on individuals are less clear, finding that stress is associated with both rational and irrational behaviour.

## Supporting information

S1 Fig The pathway of stress. Depicting how an adverse stimulus, either psychological or physiological, affects the human body. In both cases the sympathetic nervous system is activated leading to an endocrinal response (increased cortisol, but also increased heart rate, breathing rate and other unconscious physiological reactions).
(TIF)

S2 Fig Price series. Ten price series presented to participants in accordance with the predetermined sequences. Solid top and bottom lines indicate maximum range of allowable intervals.
(TIF)

S3 Fig Prediction interval screen. (TIF)

S1 Appendix Appendix materials. (PDF)

## References

[1] Holt CA, Laury SK. Risk aversion and incentive effects. American Economic Review 2002; 92 (5):1644–55. https://www.jstor.org/stable/3083270. doi: 10.1257/000282802762024700

[2] Costa P, McCrae R. Personality in adulthood: A five-factor theory perspective. New York: The Guildford Press; 2003.

[3] Coates JM, Herbert J. Endogenous steroids and financial risk taking on a London trading floor. Proceedings of the National Academy of Sciences of the United States of America 2008; 105: 6167–72. doi: 10.1073/pnas.0704025105 18413617 PMC2329689

[4] Fama EF. Efficient Capital Markets: A Review of Theory and Empirical Work. The Journal of Finance 1970; 25 (2): 383–417. doi: 10.1111/j.1540-6261.1970.tb00518.x

[5] Fama EF. Efficient Capital Markets: II. The Journal of Finance 1991; XLVI (5): 1575–1617. doi: 10.1111/j.1540-6261.1991.tb04636.x

[6] Campbell JY, Lo AW, Mackinlay AC. The econometrics of financial markets. Princeton University press 1997; 1955- Princeton, N.J.

[7] Huberman G, Regev T. Contagious Speculation and a Cure for Cancer: A Nonevent That Made Stock Prices Soar. The Journal of Finance 2001; 56 (1); 387–396. doi: 10.1111/0022-1082.00330

[8] Schlag KH, van der Weele JJ. A method to elicit beliefs as most likely intervals. Judgment and Decision Making 2015; 10 (5): 456–68. http://decisionsciencenews.com/sjdm/journal.sjdm.org/15/15402/jdm15402.pdf

[9] Daniel K, Titman S. Market Efficiency in an Irrational World. Cambridge, MA: National Bureau of Economic Research; 2000.

[10] Johnson DDP, Fowler JH. The evolution of overconfidence. Nature 2011; 477: 317–20. doi: 10.1038/nature10384 21921915

[11] Barber BM, Odean T. Boys will be boys: Gender, overconfidence, and common stock investment. Quarterly Journal of Economics 2001; 116 (1): 261–92. doi: 10.1162/003355301556400

[12] Grinblatt M, Keloharju M. Sensation seeking, overconfidence, and trading activity. Journal of Finance 2009; 64 (2): 549–78. doi: 10.1111/j.1540-6261.2009.01443.x

[13] Durand RB, Limkriangkrai M, and Fung L. The Behavioral Basis of Sell-Side Analysts’ Herding. Journal of Contemporary Accounting and Economics 2014; 10 (3): 176–90. doi: 10.1016/j.jcae.2014.08.001

[14] Juergens JL, Lindsey L. Getting out Early: An Analysis of Market Making Activity at the Recommending Analyst’s Firm. The Journal of Finance 2009; Vol. 64 (5): 2327–2359. https://www.jstor.org/stable/27735173. doi: 10.1111/j.1540-6261.2009.01502.x

[15] Jegadeesh N, Kim W. Do Analysts Herd? An Analysis of Recommendations and Market Reactions. The Review of Financial Studies 2010; 23 (2): 901–37. doi: 10.1093/rfs/hhp093

[16] Olsen RA. Implications of Herding Behavior for Earnings Estimation, Risk Assessment, and Stock Returns. Analysts Journal 1996; Vol. 52 (4): 37–41. https://www.jstor.org/stable/4479932.

[17] Prechter RR. Unconscious Herding Behavior as the Psychological Basis of Financial Market Trends and Patterns. The Journal of Psychology and Financial Markets 2001; 2 (3): 120–25. doi: 10.1207/S15327760JPFM0203_1

[18] Paese PW, Sniezek JA. Influences on the appropriateness of confidence in judgment: Practice, effort, information, and decision-making. Organizational Behavior and Human Decision Processes 1991; 48: 100–30. doi: 10.1016/0749-5978(91)90008-H

[19] Hirshleifer D. Investor psychology and asset pricing. Journal of Finance 2001; 56: 1533–97. doi: 10.1111/0022-1082.00379

[20] Soll JB, Klayman J. Overconfidence in Interval Estimates. Journal of Experimental Psychology Learning Memory and Cognition 2004; 30: 299–314. doi: 10.1037/0278-7393.30.2.299 14979805

[21] De Bondt WFM. A portrait of the individual investor. European Economic Review 1998;42 (3-5): 831–44. doi: 10.1016/S0014-2921(98)00009-9

[22] Glaser M, Langer T, Weber M. True overconfidence in interval estimates: Evidence based on a new measure of miscalibration. Journal of Behavioral Decision Making 2013; 26: 405–17. doi: 10.1002/bdm.1773

[23] Hoffmann AOI, Post T. How does investor confidence lead to trading? Linking investor return experiences, confidence, and investment beliefs. Journal of Behavioral and Experimental Finance 2016; 12: 65–78. doi: 10.1016/j.jbef.2016.09.003

[24] Stankov L, Crawford JD. Self-confidence and performance on tests of cognitive abilities. Intelligence 1997; 25: 93–109. doi: 10.1016/S0160-2896(97)90047-7

[25] Deuter CE, Kuehl LK, Blumenthal TD, Schulz A, Oitzl MS, Schachinger H. Effects of cold pressor stress on the human startle response. PLoS ONE 2012; 7:e49866. doi: 10.1371/journal.pone.0049866 23166784 PMC3499498

[26] Goldstein DS. Stress-induced activation of the sympathetic nervous system. Bailliere’s Clinical Endocrinology and Metabolism 1987;1:253–78. doi: 10.1016/S0950-351X(87)80063-0 3327494

[27] Ferracuti S, Seri S, Mattia D, Cruccu G. Quantitative EEG modifications during the Cold Water Pressor Test: Hemispheric and hand differences. International Journal of Psychophysiology 1994;17: 261–8. doi: 10.1016/0167-8760(94)90068-X 7806469

[28] Minkley N, Schröder TP, Wolf OT, Kirchner WH. The socially evaluated cold-pressor test (SECPT) for groups: Effects of repeated administration of a combined physiological and psychological stressor. Psychoneuroendocrinology 2014; 45: 119–27. doi: 10.1016/j.psyneuen.2014.03.022 24845183

[29] Seals DR. Sympathetic activation during the cold pressor test: Influence of stimulus area. Clinical Physiology 1990;10:123–9. doi: 10.1111/j.1475-097X.1990.tb00246.x 2318023

[30] Porcelli AJ. An alternative to the traditional cold pressor test: The Cold Pressor Arm Wrap. Journal of Visualized Experiments 2014; 83: p.e 50849. doi: 10.3791/50849 24457998 PMC4089407

[31] Lighthall NR, Mather M, Gorlick MA. Acute stress increases sex differences in risk seeking in the Balloon Analogue Risk Task. PLoS ONE 2009; 4: e6002. doi: 10.1371/journal.pone.0006002 19568417 PMC2698212

[32] Lighthall NR, Sakaki M, Vasunilashorn S, Nga L, Somayajula S, Chen EY. Gender differences in reward-related decision processing under stress. Social Cognitive and Affective Neuroscience 2012; 7: 476–84. doi: 10.1093/scan/nsr026 21609968 PMC3324572

[33] Cettolin E, Dalton PS, Kop WJ, Zhang W. Cortisol meets GARP: The effect of stress on economic rationality. Experimental Economics 2020; 23: 554–74. doi: 10.1007/s10683-019-09624-z

[34] Porcelli AJ, Delgado MR. Acute stress modulates risk taking in financial decision making. Psychological Science 2009; 20: 278–83. doi: 10.1111/j.1467-9280.2009.02288.x 19207694 PMC4882097

[35] Nosić A, Weber M. How riskily do I invest? The role of risk attitudes, risk perceptions, and overconfidence. Decision Analysis 2010; 7: 282–301. doi: 10.1287/deca.1100.0178

[36] Durand RB, Newby R, Tant K, Trepongkaruna S. Overconfidence, overreaction and personality. Review of Behavioral Finance 2013; 5: 104–33. doi: 10.1108/RBF-07-2012-0011

[37] Schaefer PS, Williams CC, Goodie AS, Campbell WK. Overconfidence and the Big Five. Journal of Research in Personality 2004; 38: 473–80. doi: 10.1016/j.jrp.2003.09.010

[38] Meier C, De Mello L. Investor Overconfidence in Experimental Asset Markets across Market States. Journal of Behavioral Finance 2020; 21 (4): 369–384. doi: 10.1080/15427560.2019.1692845

[39] List JA, Sadoff S, Wagner M. So you want to run an experiment, now what? Some simple rules of thumb for optimal experimental design. Experimental Economics 2011; 14 (439): 439–57. doi: 10.1007/s10683-011-9275-7

[40] Faul F, Erdfelder E, Lang A-G, Buchner A. G*Power 3: A flexible statistical power analysis program for the social, behavioral, and biomedical sciences. Behavior Research Methods 2007; 39 (2): 175–191. doi: 10.3758/BF03193146 17695343

[41] Faul F, Erdfelder E, Buchner A, Long A. Statistical power analyses using G*Power 3.1: Tests for correlation and regression analyses. Behavior Research Methods 2009; 41 (4): 1149–1160. doi: 10.3758/BRM.41.4.1149 19897823

[42] Chen DL, Schonger M, Wickens C. oTree-An open-source platform for laboratory, online, and field experiments. Journal of Behavioral and Experimental Finance 2016; 9: 88–97. doi: 10.1016/j.jbef.2015.12.001

[43] Benet-Martínez V, John OP. *Los Cinco Grandes* across cultures and ethnic groups: Multitrait-multimethod analyses of the Big Five in Spanish and English. Journal of Personality and Social Psychology 1998: 729–50. 9781409 10.1037//0022-3514.75.3.729

[44] John OP, Donahue E. M., Kentle RL. The Big Five Inventory–Versions 4a and 54. Berkeley, CA: University of California, Berkeley, Institute of Personality and Social Research; 1991.

[45] John OP, Naumann LP, Soto CJ. Paradigm shift to the integrative Big Five Trait Taxonomy: History, measurement, and conceptutal issues. In: John OP, Robins RW, Pervin LA, editors. Handbook of personality: Theory and research, vol. 3, New York, NY: Guildford Press; 2008, pp. 114–58.

[46] Budescu DV, Du N. Coherence and consistency of investors’ probability judgments. Management Science 2007; 53 (11): 1731–44. doi: 10.1287/mnsc.1070.0727

[47] De Bondt WFM. Betting on trends: Intuitive forecasts of financial risk and return. International Journal of Forecasting 1993; 9: 355–71. doi: 10.1016/0169-2070(93)90030-Q

[48] Glaser M, Langer T, Reynders J, Weber M. Framing effects in stock market forecasts: The difference between asking for prices and asking for returns. Review of Finance 2007; 11 (2): 325–57. doi: 10.1093/rof/rfm008

