# Supplementary figures and images for: Predicting price intervals under exogenously induced stress
Source: PLoS One. 2021 Sep 23;16(9):e0255038. doi: 10.1371/journal.pone.0255038 (PMC8460030; doi:10.1371/journal.pone.0255038)

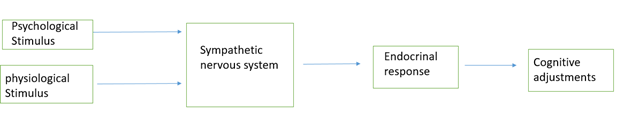

Supplement: S1 Fig — Depicting how an adverse stimulus, either psychological or physiological, affects the human body. In both cases the sympathetic nervous system is activated leading to an endocrinal response (increased cortisol, but also increased heart rate, breathing rate and other unconscious physiological reactions). (TIF) [file pone.0255038.s001.tif]

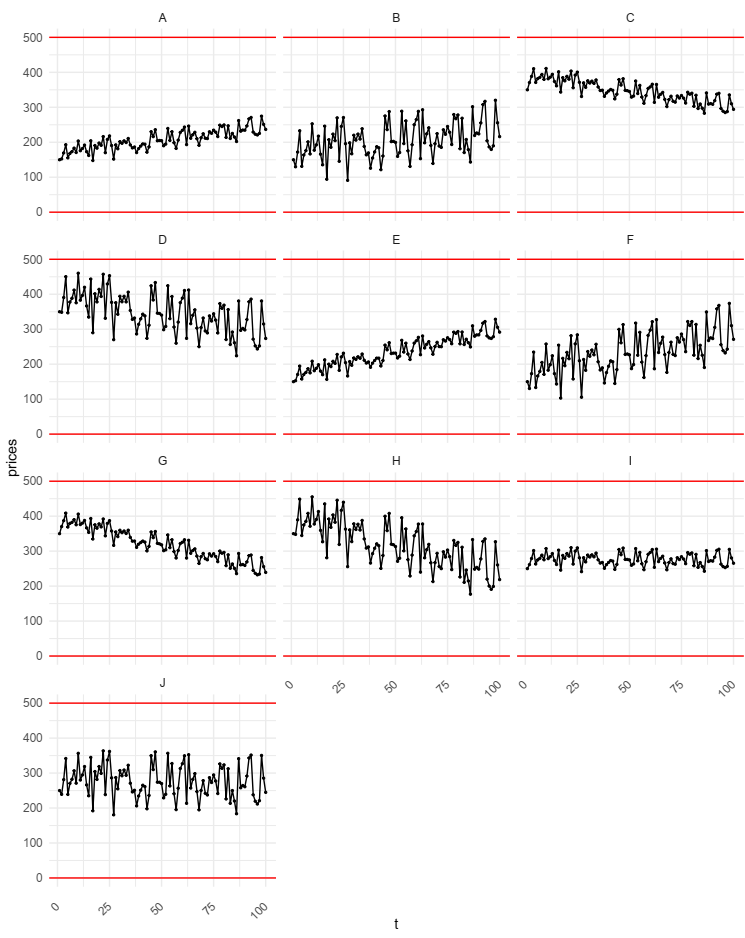

Supplement: S2 Fig — Ten price series presented to participants in accordance with the predetermined sequences. Solid top and bottom lines indicate maximum range of allowable intervals. (TIF) [file pone.0255038.s002.tif]

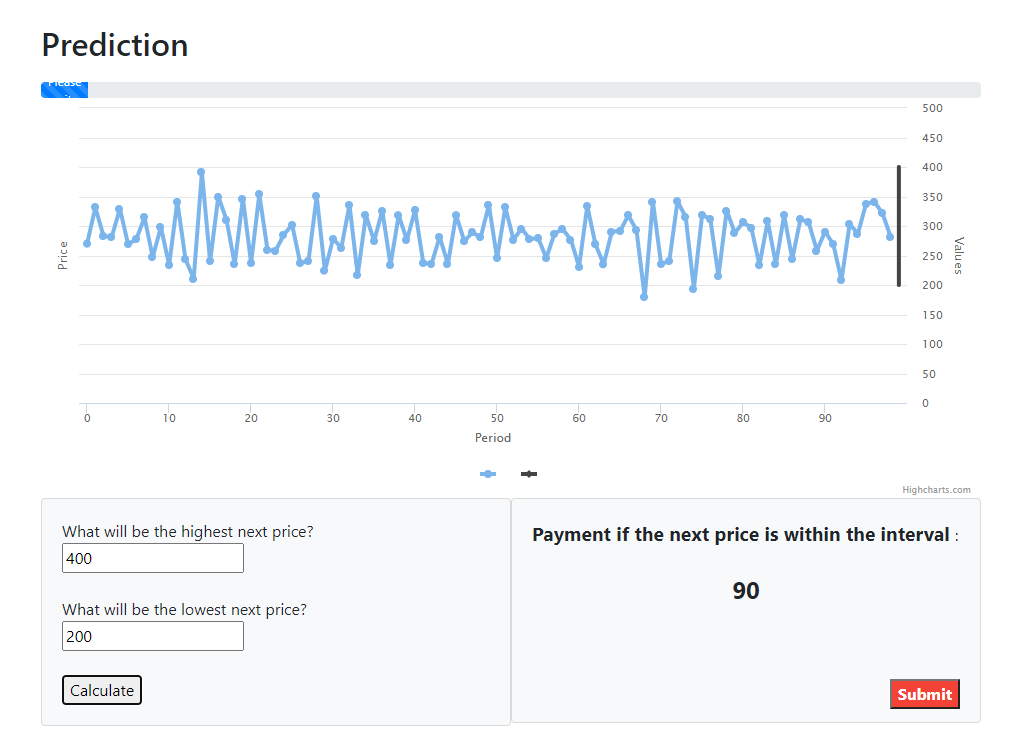

Supplement: S3 Fig — (TIF) [file pone.0255038.s003.tif]
