# Supplementary material for: Predicting price intervals under exogenously induced stress
Source: PLoS One. 2021 Sep 23;16(9):e0255038. doi: 10.1371/journal.pone.0255038 (PMC8460030; doi:10.1371/journal.pone.0255038)
Supplement: S1 Appendix — (PDF) [file pone.0255038.s004.pdf]

# Prediction Intervals

Rewrite, with the upper bound represented as $P_l + \delta$:

$$\mathbb{E}(\Pi) = \Big(\Phi(P_l + \delta) - \Phi(P_l)\Big)\Big(1 - \frac{\delta}{M}\Big)B$$

Choose $P_l$ and $\delta$ to maximise:

$$\frac{\partial \mathbb{E}(\Pi)}{\partial P_l} = \Big(\phi(P_l + \delta) - \phi(P_l)\Big)\Big(1 - \frac{\delta}{M})B = 0$$

$$\phi(P_l + \delta) = \phi(P_l)$$

$$|P_l + \delta| = |P_l| \text{ the distribution is centered around } \frac{\delta}{2}$$

The mid point of $\delta$ is:

$$\underbrace{P_l + \frac{\delta}{2}}_{\text{lower bound plus } \frac{\delta}{2}} = m = \underbrace{P_l + \delta - \frac{\delta}{2}}_{\text{upper bound minus} \frac{\delta}{2}},$$

which implies that

$$P_l = m - \frac{\delta}{2}, \qquad \text{and}$$

$$P_l + \delta = m + \frac{\delta}{2} = -(m - \frac{\delta}{2}),$$

Thus

$$|P_l| = |P_l + \delta|$$

$$\frac{\partial \mathbb{E}(\Pi)}{\partial \delta} = \Big(\phi(P_l + \delta)\Big)(1 - \frac{\delta}{M})B - \Big(\Phi(P_l + \delta) - \Phi(P_l)\Big)\frac{B}{M} = 0$$

$$\Big(\phi(P_l + \delta)\Big)(1 - \frac{\delta}{M})B = \Big(\Phi(P_l + \delta) - \Phi(P_l)\Big)\frac{B}{M}$$

$$\Big(\phi(P_l + \delta)\Big)(1 - \frac{\delta}{M})\frac{BM}{B} = \Big(\Phi(P_l + \delta) - \Phi(P_l)\Big)$$

$$M - \delta = \frac{\Big(\Phi(P_l + \delta) - \Phi(P_l)\Big)}{\phi(P_l + \delta)}$$

$$\delta = M - \frac{\Big(\Phi(P_l + \delta) - \Phi(P_l)\Big)}{\phi(P_l + \delta)}$$

$$M - \delta - \frac{\Big(\Phi(P_l + \delta) - \Phi(P_l)\Big)}{\phi(P_l + \delta)} = 0$$

There is a unique optimal interval of size $\delta$ for any choice of $P_l$, given $M$, $\mu = P_0 + \beta_1 t + \beta_2 P_t$ and $\epsilon_t \sim N(0, \sigma^2)$.

# Instruction Screens 7

## Screen 1 8

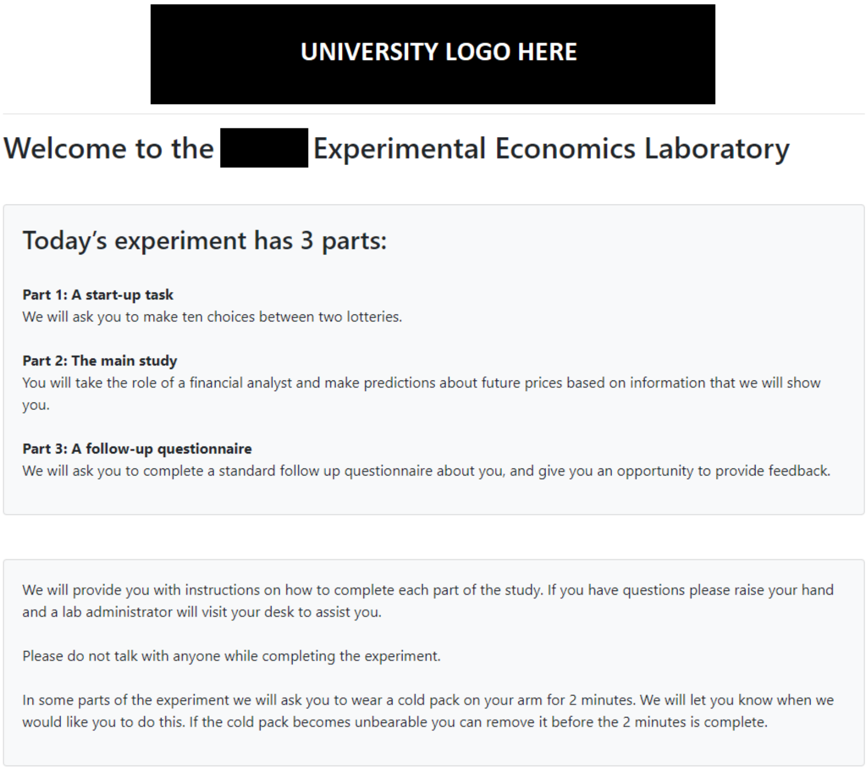


9

# Screen 2

## Part 1

### Individual Task

On the next page we will ask you to make ten decisions between 'Option A' and 'Option B'.
For example:

- Option A could be $3.00 with probability 3/10 and $0.50 with probability 7/10
- Option B could be $2.00 with probability 3/10 and $1.75 with probability 7/10

| | | | | |
|---|---|---|---|---|
| **3** | 3/10 of $2.00, 7/10 of $1.60 | Option A | Option B | 3/10 of $3.85, 7/10 of $0.10 |

### How choices affect earnings:

**Stage 1**
One of your ten choices will be chosen randomly.
Each of the 10 decisions are equally likely.

**Stage 2**
Another random draw from 1-10 will then be made to determine your payment.
Each number is equally likely.

For example, if you chose Option A $3.00 with probability 3/10 and $0.50 with probability (7/10):

- If the random draw is 1,2 or 3 you will earn $3.00.
- If the random draw is 4,5,6,7,8, 9 or 10 you will earn $0.50.

**Summary**
Even though you will make ten decisions, only one of these will end up affecting your earnings today and you will not know in advance which decision will be used.
Each decision has an equal chance of being used in the end and these random numbers will be different for each participant in today's session.

Next-->

## Screen 3

### Individual Task

**Now please look at the empty circles in the centre of your computer screen. You will have to make a decision, Option A or Option B, by clicking one of the two circles. Now you may begin making your choices.**

| # | Option A | choice | Option B |
|---|---|---|---|
| **1** | 1/10 of $2.00, 9/10 of $1.60 | ○ Option A ○ Option B | 1/10 of $3.85, 9/10 of $0.10 |
| **2** | 2/10 of $2.00, 8/10 of $1.60 | ○ Option A ○ Option B | 2/10 of $3.85, 8/10 of $0.10 |
| **3** | 3/10 of $2.00, 7/10 of $1.60 | ○ Option A ○ Option B | 3/10 of $3.85, 7/10 of $0.10 |
| **4** | 4/10 of $2.00, 6/10 of $1.60 | ○ Option A ○ Option B | 4/10 of $3.85, 6/10 of $0.10 |
| **5** | 5/10 of $2.00, 5/10 of $1.60 | ○ Option A ○ Option B | 5/10 of $3.85, 5/10 of $0.10 |
| **6** | 6/10 of $2.00, 4/10 of $1.60 | ○ Option A ○ Option B | 6/10 of $3.85, 4/10 of $0.10 |
| **7** | 7/10 of $2.00, 3/10 of $1.60 | ○ Option A ○ Option B | 7/10 of $3.85, 3/10 of $0.10 |
| **8** | 8/10 of $2.00, 2/10 of $1.60 | ○ Option A ○ Option B | 8/10 of $3.85, 2/10 of $0.10 |
| **9** | 9/10 of $2.00, 1/10 of $1.60 | ○ Option A ○ Option B | 9/10 of $3.85, 1/10 of $0.10 |
| **10** | 10/10 of $2.00, 0/10 of $1.60 | ○ Option A ○ Option B | 10/10 of $3.85, 0/10 of $0.10 |

Next

# Screen 4 14

## The Experiment

In this experiment, you will take on the role of a financial analyst. Today's task is to examine a set of graphs of the last 100 periods of a stock's price and to predict the interval within which the next price will occur.

**The prices will look like this:**

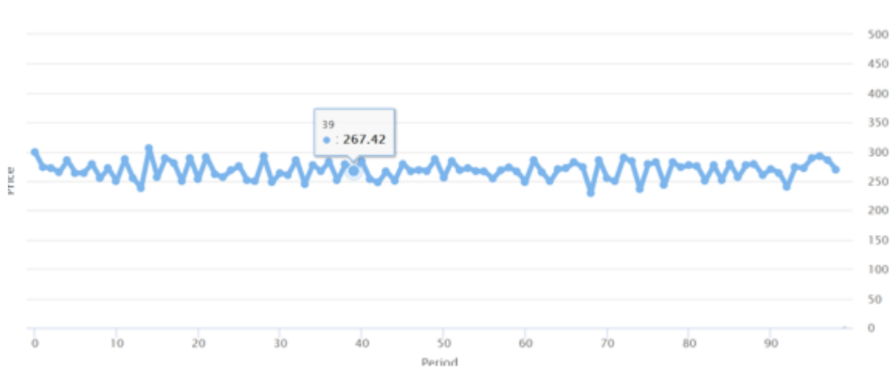


To get information on an individual time period's price, hover over with the cursor on a point on the graph, a point is represented by a circle.

Each price series has been generated using a formula that involves some randomness.

15

## Screen 5 16

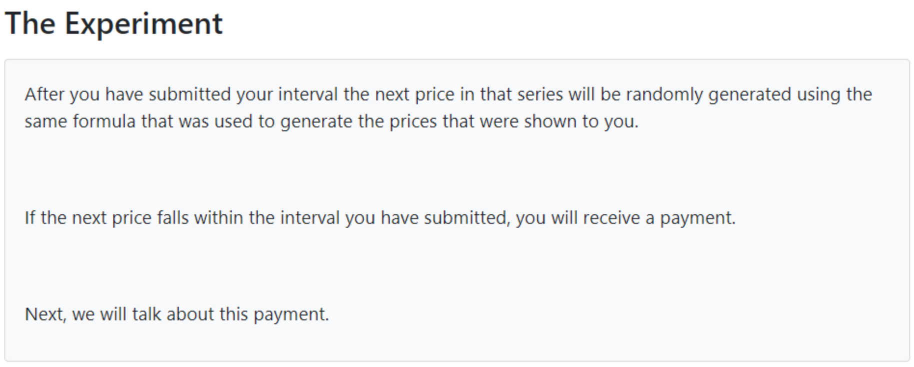


17

## Screen 6 18

### The Experiment

For each price series the lowest price to construct your lower bound is 0 and the highest price to construct your upper bound will be indicated on the screen.

The range of the interval is defined as the highest price minus the lowest price that you submitted.

The maximum amount you can earn today is $42.

**The maximum points you can earn if the next price falls within the range of your interval is 100 points. 100 points you will earn will be converted to 70 cents at the end of the experiment.**

- The amount of points you will earn depends on the range of the interval.
- A smaller interval range will result in more points.
- A larger interval range will result in fewer points.

**If the next price does not fall within the interval you submit you will receive nothing.**

We will provide you with an on-screen calculator to compute the amount of points you will receive if the next price falls within your interval range; this will be discussed next.

19

## Screen 7 20

### The Experiment

To assist you with making your decisions today we will provide you with a special on-screen calculator which will show the amount you will earn if the price falls within the interval range. You can try out different interval ranges using this calculator before submitting your decision.

**This is what on-screen calculator looks like:**

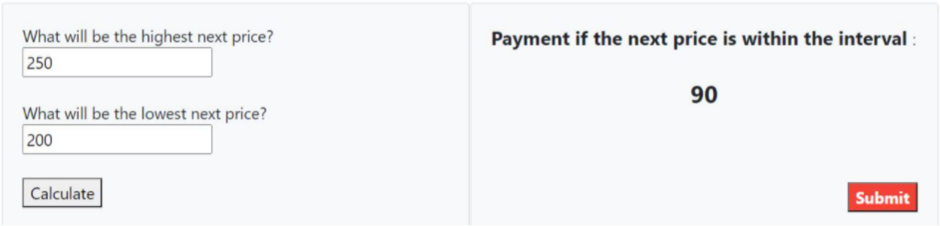


When you press **calculate**, it will show you the payment if the next price falls within the interval range .

Remember that if the price does not fall within the interval you submit you will earn nothing.

By pressing submit, you are submitting your final answer and you will move on to the next round.

**You can try out this calculator in the practice rounds which will start shortly.**

21

## Screen 8 22

### The Experiment

Your interval will also be shown on the graph via a black straight line. Please see an example of this below. Every time you click calculate it will adjust the black straight line to your new interval.

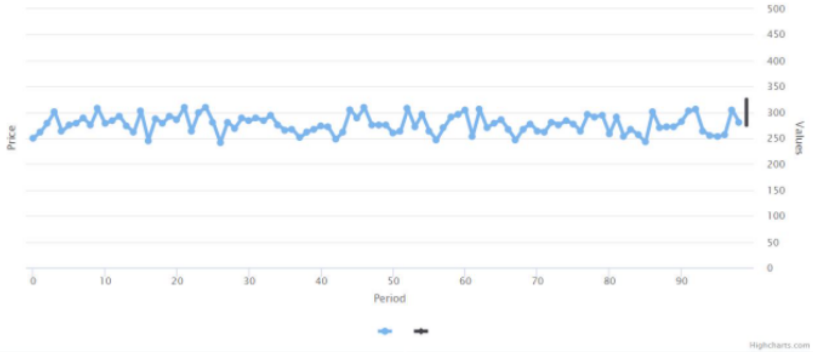


What will be the highest next price?
325

What will be the lowest next price?
275

Calculate

**Payment if the next price is within the interval** :

**90**

**Submit**

You will see many sets of prices during today's study and submit a number of prediction intervals. We will randomly generate the next price in each series, and you will learn the results of each of your price interval predictions at the end of the experiment.

At the end of the session, after you complete the final questionnaire, you will be directed to the screened desk at the front of the room to receive your cash payment. 23

## Screen 9 24

### The Treatment

We will ask you to wear an arm wrap that contains a gel pack for 2 minutes at the start of each block of 15 price series you will see.

**Sometimes the cold pack will be cold other times it will not.**

If the arm wrap becomes unbearable at any point you may remove it but please press pause.

Please watch the video down below on how to apply the arm wrap on your arm

25

# Comprehension Test

## Quiz Part 1

**Now answer the below questions, this is to test your understanding of the task. Until you have selected all the correct answer you can not continue.**

1. If the next price in the time-series is 330 dollars and you have selected 100 dollars for your lower bound and 225 dollars for your upper bound. Will you receive any payment?

- Yes
- No

2. If the next price in the time-series is 250 dollars and you have selected a lower bound of 200 dollars and an upper bound of 290 dollars. Will you receive payment?

- No
- Yes

3. Does the upper bound have to be larger or smaller then the lower bound?

- Larger
- Smaller

4. If the next price in the time series fell inside your prediction interval, which of the following intervals would yield the higher payoff?

- 200 - 300
- 225 - 275

5. Can you take off the arm wrap before 2 minutes is completed?

- Yes
- No

**Next you will complete 3 practice rounds.**

Next

# Practice Round 1 28

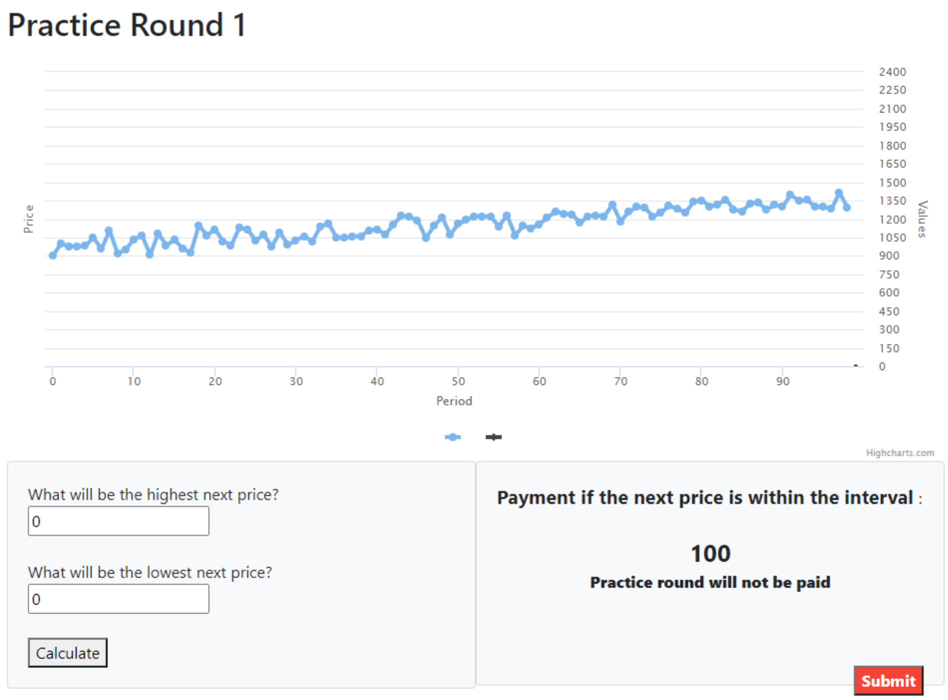


29

# Practice Round 2 30

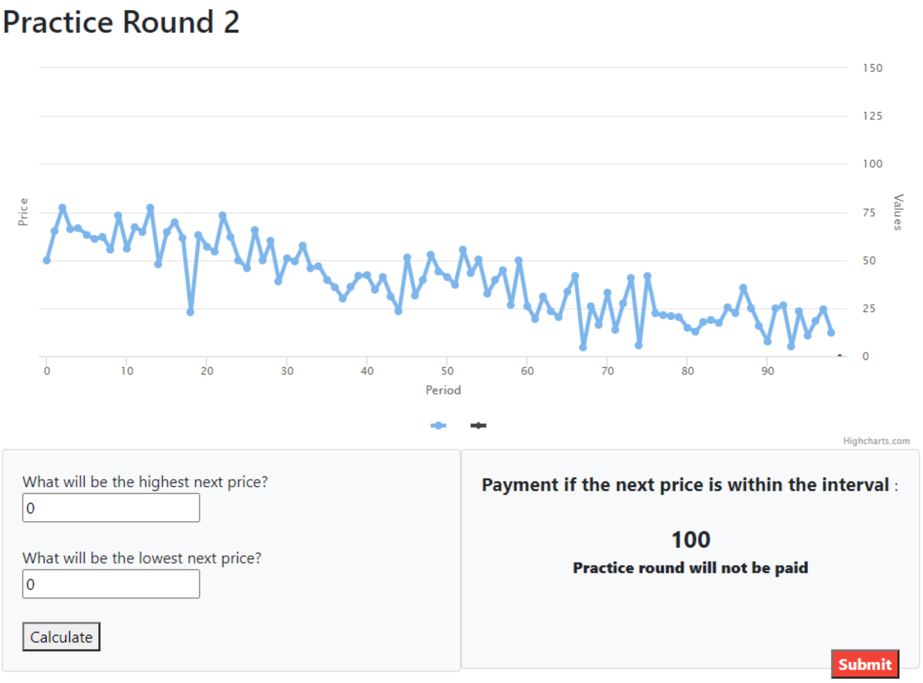


31

# Practice Round 3

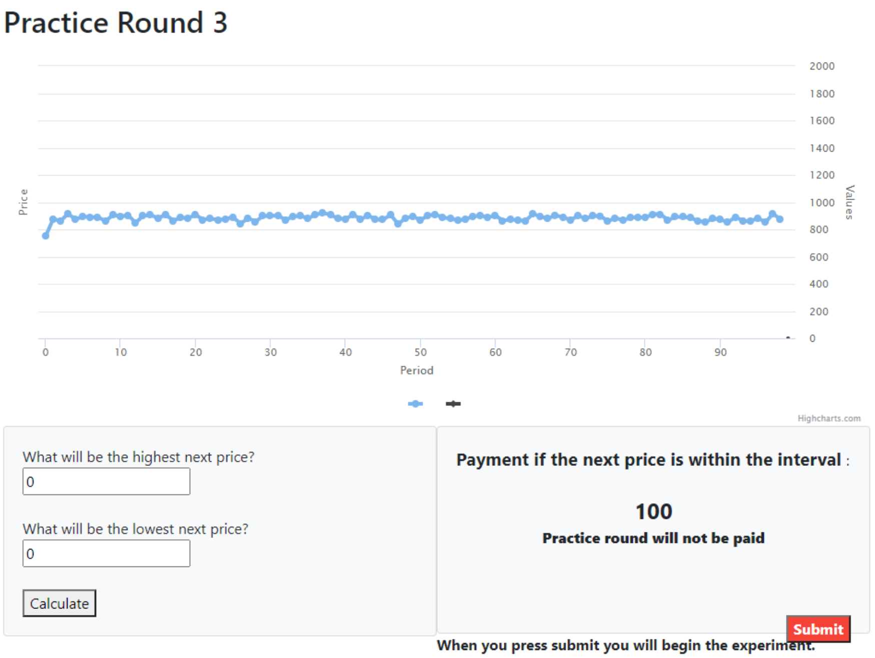

# Comprehension Test Part 2 34

## Quiz Part 2

In one or two sentences, please describe your strategy to maximise your earnings.

Next

35

# Screens that are in the main experiment

## Application of the CPAW screen

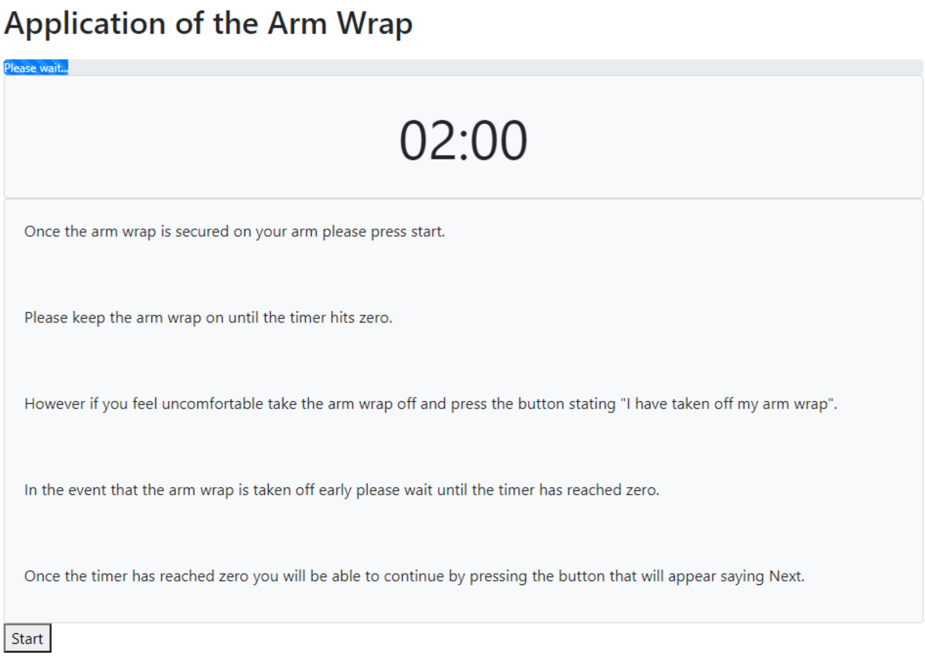

## Example of the experiment round screen (60 similar screens)

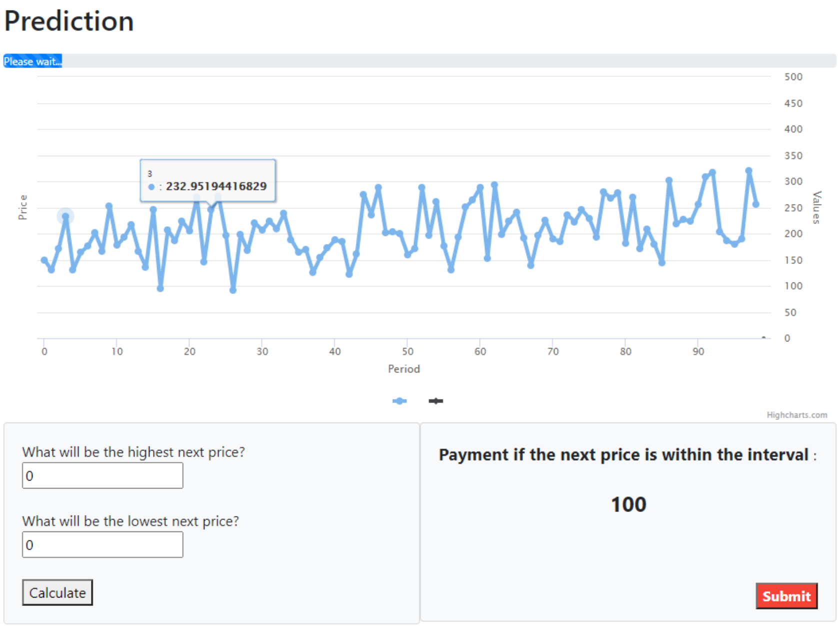

# Demographic Questions (following 60 decisions) 41

## About you

**In this section we do not require you to answer any of the questions below.**
**If you choose not to answer any questions please select on the drop-down box 'prefer not to say' from the drop down box.**

What gender do you identify with?

---------

What is your main role at Curtin? :

---------

What is your major area of study (if you are a student)? :

---------

If you are not a student what is your occupation? :

---------

What year were you born in? :

---------

Before today, how many experiments had you participated in at the Curtin Experimental Economics Lab? :

---------

Before today, had you ever participated in an economics experiment (at another university/ in a class)? :

---------

Next 42

# Summary of Results (end of experiment) 43

## Summary of Results

| | Results |
|---|---|
| **Show up fee** | |
| **Individal Task** | $2.50 |
| **Total Points Won** | 0 points |
| **Points Dollar Value** | $0 |
| **Total Payment** | $5 |

| Round | Correct Answer | Lower | Upper | won | Points won |
|---|---|---|---|---|---|
| **1** | 0.0 | 0.0 | 0.0 | "No" | 0 |
| **2** | 0.0 | 0.0 | 0.0 | "No" | 0 |
| **3** | 0.0 | 0.0 | 0.0 | "No" | 0 |

Next

44
